# New Approach for Targeting Small-Molecule Candidates for Intrinsically Disordered Proteins

**DOI:** 10.3390/mps8060150

**Published:** 2025-12-10

**Authors:** Milan Senćanski

**Affiliations:** Laboratory of Bioinformatics and Computational Chemistry, Institute of Nuclear Sciences Vinca, National Institute of the Republic of Serbia, University of Belgrade, 11001 Belgrade, Serbia; sencanski@vin.bg.ac.rs

**Keywords:** drug discovery, virtual screening, proteins, bioinformatics, tau protein, drug bank

## Abstract

Intrinsically disordered proteins (IDPs), such as the Alzheimer’s-associated tau protein, pose challenges for conventional drug discovery. This study applied the Informational Spectrum Method for Small Molecules (ISM-SM), a computational technique utilizing electron–ion interaction potentials (EIIPs), to identify potential tau modulators. Characteristic interaction frequencies derived from known ligands and conserved mammalian tau sequences were used to screen DrugBank and the COCONUT natural product database. The screening identified approved drugs previously reported to indirectly influence tau pathology or Alzheimer’s disease pathways, alongside natural products including Bryostatin-14, which is known to modulate kinases involved in tau phosphorylation. These findings suggest that ISM-SM can serve as an in silico tool to identify candidate small molecules, including repurposed drugs and natural products, with potential relevance to tau function and pathology, complementing other IDP drug discovery strategies.

## 1. Introduction

Intrinsically disordered proteins (IDPs) play essential roles in a variety of biological processes and have been associated with numerous diseases, including neurodegenerative disorders and viral infections [1,2]. In contrast to structured proteins, which contain stable binding pockets, IDPs exist as dynamic conformational ensembles and represent particularly challenging targets in small-molecule drug discovery. Several small molecules have been identified as IDP ligands, usually by binding to transient interaction sites or as modulators of their dynamic conformational states. Notable examples include epigallocatechin gallate (EGCG) for α-synuclein [3], Phenothiazine for tau [4], and various compounds targeting viral nucleocapsid proteins [5]. IDPs’ inherent flexibility requires specialized experimental and computational methods to identify their ligands.

Conventional structure-based drug development methods are not as successful with IDPs because the binding sites are poorly defined. Similarly, high-throughput virtual screening methods struggle with IDP dynamic and heterogeneous nature. They may call for alternate techniques such as ensemble docking [6,7,8,9,10,11], molecular dynamics (MD) simulations [12,13,14,15,16,17,18,19,20], and machine learning models trained using IDP–ligand interactions [16,21,22,23,24,25]. Experimental methods, including nuclear magnetic resonance (NMR) spectroscopy, surface plasmon resonance (SPR), and fluorescence-based assays, can also help demonstrate weak, transient interactions [26]. Therefore, computational and biophysical methods are crucial for discovering and optimizing small-molecule ligands.

The standard new drug development process typically involves hit identification, lead optimization, preclinical testing, and clinical trials. The cost of bringing a drug to market follows the industry standard, averaging USD 1.39–2.87 billion over 10–15 years, with high attrition rates due to the complexity of validating functional effects and ensuring specificity [27]. Due to the additional complexity of IDP–ligand interactions, this process is more challenging, and the introduction of various in silico methods is required.

The ISM-SM (Informational Spectrum Method for Small Molecules) method offers a distinct approach compared to traditional High-Throughput Screening (HTPS) methods for finding small-molecule candidates for disordered proteins, specifically by analyzing long-range interaction potential rather than relying solely on structure [28]. ISM-SM can put molecular structures into a frequency spectrum, enabling it to identify the compatible interaction frequencies for small molecules and target proteins, predicting biological activity. Our previous ISM-SM studies have successfully identified biologically active ligands for specific protein binding sites [29,30]. ISM-SM has also been utilized to discover binders towards proteins associated with emerging viral threats, such as SARS-CoV-2. An example of this kind is the determination of the interaction frequencies between small molecules that have been shown to be beneficial against viral proteins, predicting drugs that could be repurposed for COVID-19, and significantly accelerating the drug discovery process in response to a pressing public health emergency [31,32,33]. This study explores the application of ISM-SM for identifying potential therapeutic candidates against selected disease targets. We focus on human tau protein candidates, one of the two hallmark proteins of Alzheimer’s disease (AD) [34].

## 2. Materials and Methods

### 2.1. Databases

The sequence of Human microtubule-associated protein tau (P10636) was taken from the UniProt database (www.uniprot.org, accessed on 30 October 2025) [35]. Tau proteins from other mammals were also downloaded:

- O02828 *Capra hircus*;
- P10637 *Mus musculus*;
- P19332 *Rattus norvegicus*;
- P29172 *Bos taurus*;
- P57786 *Macaca mulatta*;
- Q5S6V2 *Pongo pygmaeus*;
- Q5YCV9 *Hylobates lar*;
- Q5YCW0 *Gorilla gorilla gorilla*;
- Q5YCW1 *Pan troglodytes*;
- Q6TS35 *Spermophilus citellus*;
- Q9MYX8 *Papio hamadryas*.

For the screening of drugs for repurposing to select candidates for Microtubule-associated protein tau, 2627 approved small-molecule drugs from DrugBank [36] (http://www.drugbank.ca, accessed on 30 October 2025) were screened. The criteria for candidate selection were five reported tau protein binding drugs: DB00637 Astemizole, DB01248 Docetaxel, DB14914 Flortaucipir F-18, DB00448 Lansoprazole, and DB01229 Paclitaxel.

The COCONUT database [37] is a freely available collection of over six hundred thousand natural products, some of which may also be commercially available (https://coconut.naturalproducts.net/, accessed on 30 October 2025). The COCONUT database was downloaded, and compounds were converted to SMILES notation.

### 2.2. ISM-SM Method

In this study, we investigate the tau protein and its small-molecule ligands using the Informational Spectrum Method for Small Molecules (ISM-SM), an extension of the Informational Spectrum Method (ISM). This bioinformatics approach enables the analysis of long-range molecular interactions based on the electronic properties encoded in primary structures of biomolecules.

The ISM-SM approach consists of the following key steps:

1. Numerical Encoding of Protein Sequences: The primary amino acid sequence of the protein is converted into a numerical series by assigning each residue its corresponding electron–ion interaction potential (EIIP) value.
2. Numerical Encoding of Small Molecules: The molecular structure of a small molecule, represented in SMILES notation, is translated into a numerical sequence by mapping each atomic group to its EIIP value.
3. Informational Spectrum Calculation: The numerical sequences obtained for the protein and small molecules are transformed into informational spectra (IS) using the discrete Fourier transform (DFT). This process decomposes the sequences into frequencies and amplitudes, revealing periodicities corresponding to structural and functional motifs.
4. Cross-Spectrum (CS) Analysis: The interaction potential between the protein and small molecules is assessed by calculating the cross-spectrum, which identifies shared frequencies in their respective IS profiles. These common frequencies indicate potential sites of interaction or functional correlation.

The electron–ion interaction potential (EIIP) is a physical parameter that reflects the long-range electronic properties of biomolecules, relevant for interactions at distances ranging from 5 to 1000 Å [38,39]. EIIP values are computed using the following equations:

(1) $$W=0.25\frac{Z^{*}\times sin(1.04\pi Z^{*})}{2\pi },$$

(2) $$Z^{*}=\frac{1}{N}\sum_{i=1}^{m}n_{i}Z_{i}$$

In Equation (2), *Z*∗ is the average quasi-valence number (AQVN), *N* is the total number of atoms, *n_i_* is the number of atoms of the *i*-th type, and *Z_i_* is the valence of the *i*-th element. The EIIP values derived from Equation (1) are expressed in Rydbergs (Ry) [40,41].

The discrete Fourier transformation (DFT) used to obtain the IS is defined by

(3) $$X(n)=\sum_{m=1}^{N}x\left(m\right)e^{-i2\pi (m-1)/N},\;n=1,2,\ldots ,N/2$$

Here (3), *x*(*m*) is the *m*-th element of the input numerical sequence of length *N*, and *X*(*n*) is the resulting Fourier coefficient. The frequencies in the IS correspond to biologically relevant periodicities associated with structural or functional motifs in the sequence. To identify shared informational content between molecules, a cross-spectrum (CS) is constructed:

(4) $$C\left(j\right)=\prod_{i=1}^{N}S(i,j)$$

In Equation (4), *C*(*j*) is the *j*-th component of the CS, and *S*(*i*,*j*) is the *j*-th frequency component of the *i*-th IS. Frequencies with high amplitudes in the CS indicate conserved information relevant to biological function or molecular interaction. The amplitude reflects interaction strength, while the signal-to-noise ratio (*S/N*) provides a measure of interaction specificity.

Finally, sliding window analysis across the protein sequence allows for the localization of domains contributing to specific intermolecular recognition, enabling the identification of potential binding regions for interacting partners, including proteins and small molecules.

### 2.3. Drug Score Calculation

Drug Score (*dS*) values are calculated in DataWarrior [42]. The following descriptors required are calculated (s): druglikeness (*d*), logP, logS, Molecular Weight (MW), and four types of drug toxicity (t): primary irritation, mutagenic effects, reproductive effects, and tumorigenic effects. The druglikeness in DataWarrior is partially based on topological descriptors, fingerprints of MDL structure keys or other properties as cLogP and molecular weights, including a list of about 5300 distinct substructure fragments with associated druglikeness scores. The druglikeness is calculated using the following Equation (5), summing up score values of those fragments that are present in the molecule under investigation:

(5) $$d=\frac{\sum v_{i}}{\sqrt{n}}$$

The drug score (*dS*) is calculated according to the following formulas (6) and (7):

(6) $$dS=\prod (\frac{1}{2}+\frac{1}{2}s_{i})\cdot \prod t_{i}$$

(7) $$s_{i}=\frac{1}{1+e^{ap+b}}$$

where p corresponds to logP, logS, MW and *d*, and parameters *a* and *b* correspond to values {1, −5}, {1, 5}, {0.012, 6}, {1, 0}, respectively. The *t_i_* values are 1.0, 0.8 and 0.6 for no risk, low risk, and high risk, respectively. A detailed reference on the calculation of molecular properties can be found in the DataWarrior manual at https://openmolecules.org/properties/properties.html (accessed on 30 October 2025).

The Total Score had to incorporate long-range interaction properties, measured according to the ISM-SM signal-to-noise ratio (*S/N*) and Drug Score. As both parameters favor the higher values, the *Total Score* is therefore calculated as their product (8), i.e.,

(8) Total Score=dS×S/N

**Scheme 1:**
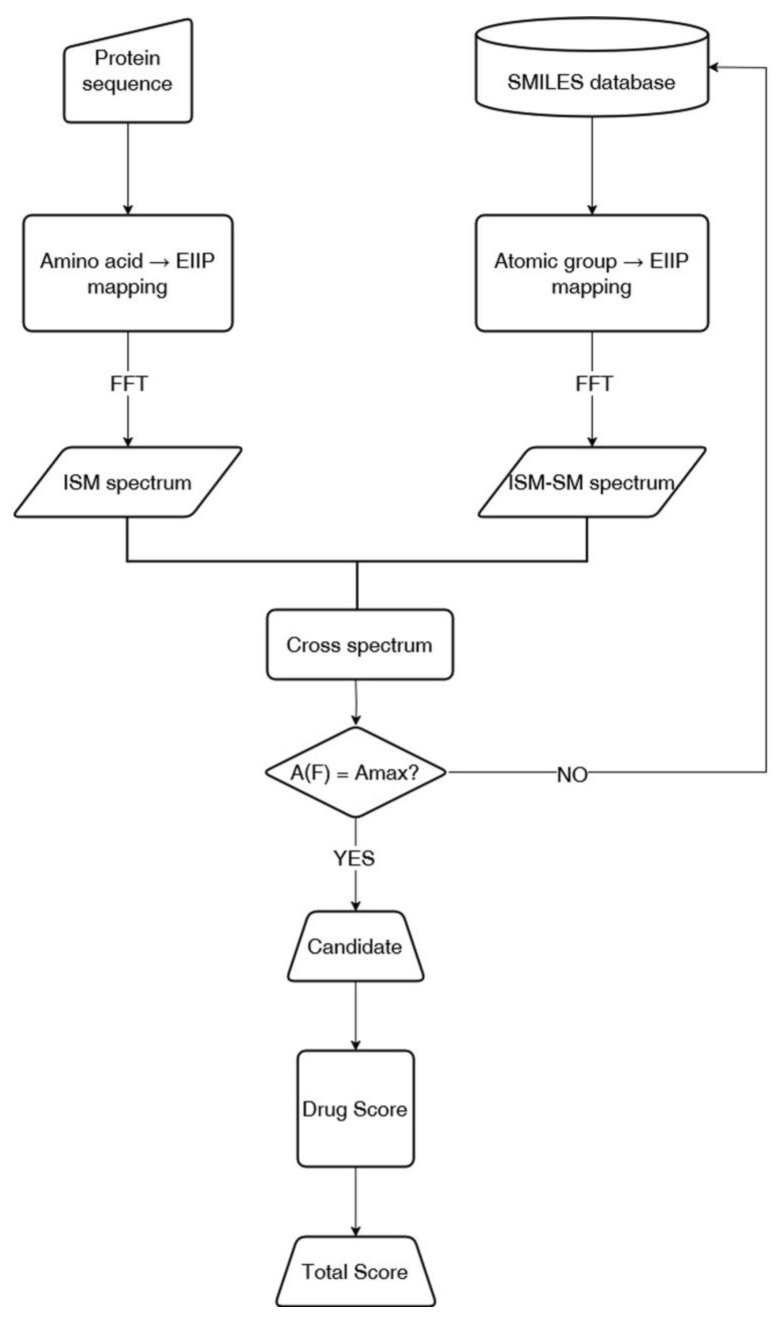
Methodology workflow. A(F)—value of the ISM amplitude at the frequency F. Amax—maximum amplitude value among all frequencies.

The full methodology workflow is presented in Scheme 1.

### 2.4. Pharmacokinetics Predictions

The pharmacokinetic properties of the compounds were calculated using the pkCSM [43] server (https://biosig.lab.uq.edu.au/pkcsm/prediction, accessed on 25 November 2025), with a total number of variables of 36. The detailed interpretation of calculated ADMET properties is available at https://biosig.lab.uq.edu.au/pkcsm/theory (accessed on 25 November 2025). In the case of non-numerical prediction results Yes/No, for further calculations, they were converted to 1 and 0, respectively. The resulting data were subjected to k-means clustering.

### 2.5. Continuous Wavelet Transform (CWT)

In this study, we use CWT to identify hotspot amino acid residues in a protein sequence at a specific ISM frequency. The detailed procedure is described in our previous work [44].

## 3. Results and Discussion

### 3.1. DrugBank Candidates

**Figure 1:**
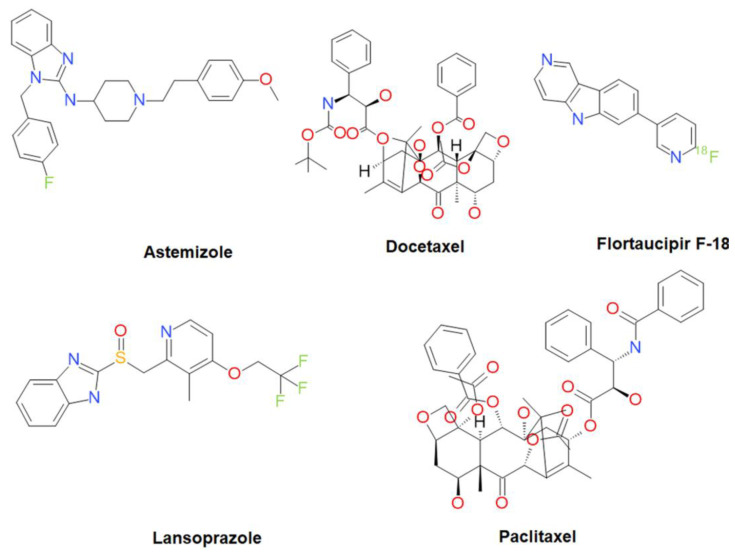
Structures of DrugBank compounds directly binding to the tau protein.

**Figure 2:**
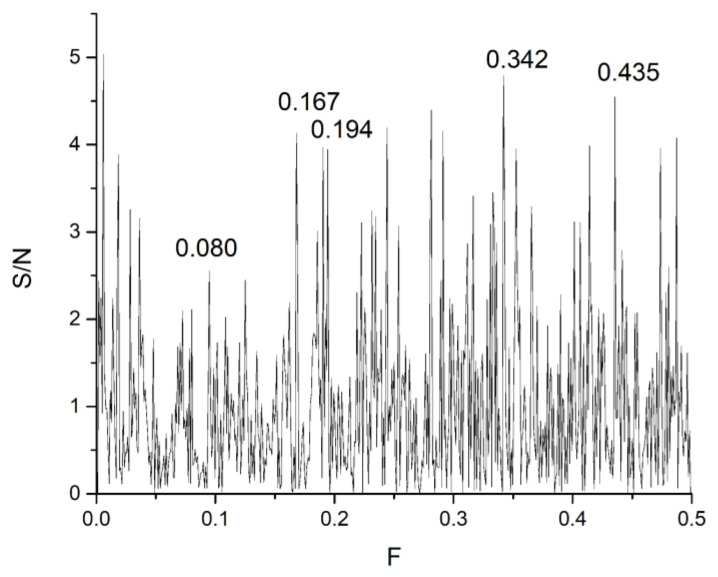
ISM spectrum of the tau protein.

**Figure 3:**
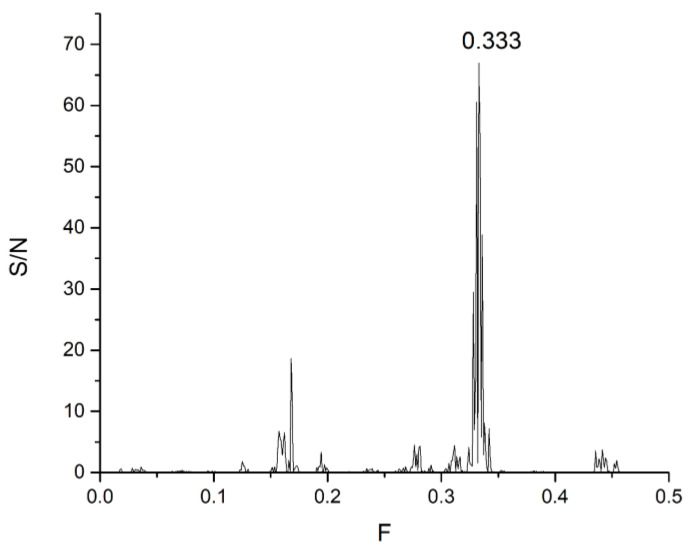
Cross-spectrum of all five tau-protein-targeting drugs from DrugBank.

**Figure 4:**
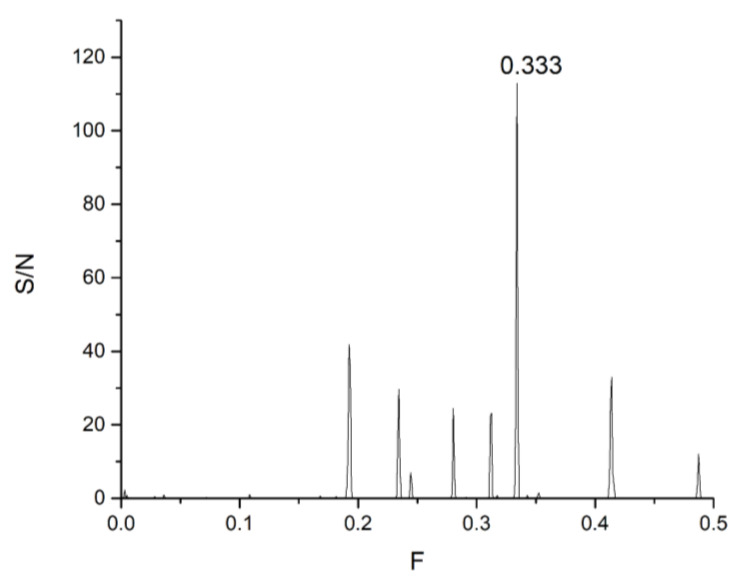
Cross-spectrum of all mammal tau proteins.

Five reported drugs from the Uniprot database targeting Microtubule-associated protein tau protein (P10636) were extracted with their structures in SMILES format (Figure 1). The structures were converted into the explicit hydrogen format, and their CS with the tau protein were calculated, along with the corresponding interaction domains (Table 1). Five frequencies, F(0.080), F(0.167), F(0.194), F(0.342) and F(0.435), were identified (Figure 2). The additional frequency from CS, including all five drugs and tau protein, was found at F(0.333) (Figure 3). Interestingly, the same frequency was obtained from the CS of all mammal tau proteins (Figure 4). This suggests an evolutionarily conserved region in the tau protein.

**Figure 5:**
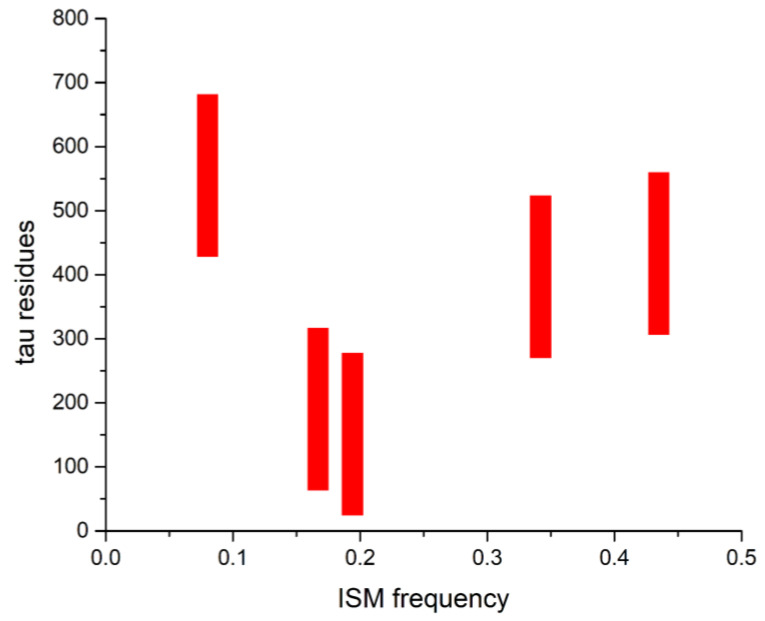
The domains in the tau protein corresponding to CS frequencies with the ligands.

Regarding the binding domains of tau protein, Astemizole, Flortaucipir F-18 and Lansoprazole bind to R3-R4 regions [45,46,47], while Docetaxel and Paclitaxel do not bind directly to tau, but β-tubulin. The calculated domains corresponding to CS frequencies agree with the literature, even indirectly, due to the partial spanning of the Microtubule-Binding (MTBD) region (244–368) [48] (Figure 5). The region corresponding to F (0.333) corresponds to the residues 494–750. This C-terminal domain is not directly responsible for microtubule binding, but rather it may modulate or influence this interaction indirectly under certain conditions, such as phosphorylation or aggregation [49].

**Figure 6:**
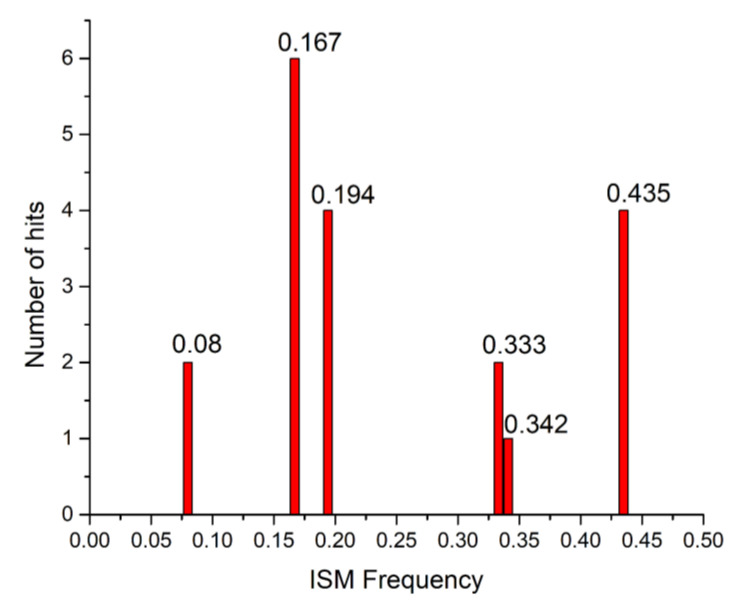
Distribution of DrugBank hit compounds at different ISM frequencies.

The 2627 approved DrugBank candidates’ structures were subjected to the exact format conversion as literature compounds and were further CS-scanned at all six frequencies. Of the candidates obtained (Table S1), 19 were already reported to indirectly affect the tau protein or AD progression (Table 2, Figure 6).

Cinacalcet was reported to play a significant role in AD. As a calcimimetic agent used for hyperparathyroidism, it modulates calcium-sensing receptors, which may influence amyloid-beta (Aβ) pathology and neuronal calcium dysregulation, both implicated in AD. It may indirectly influence tau phosphorylation by regulating calcium signaling, but direct evidence is lacking [50]. Bezafibrate is a PPAR-alpha agonist used for lipid disorders. It has been shown that bezafibrate treatment could attenuate the severity of tau pathology in the streptozotocin-intracerebroventricular-induced sporadic AD rat model [51]. Temsirolimus [52,53] and Everolimus [54,55] were reported to demonstrate neuroprotective effects in AD models by reducing tau hyperphosphorylation and promoting autophagic clearance of amyloid-β (Aβ) and tau aggregates, improving cognitive function. Desmopressin [56], a neurohypophyseal hormone analog, has been suggested to modulate amyloid aggregation, though its direct role in tau pathology remains less explored. Prednicarbate [57] (a topical corticosteroid) was identified in a drug screening study as one of the prescription drugs that may influence hyperphosphorylated tau aggregation and cytotoxicity. Roflumilast [58] ameliorates cognitive deficits in AD mice by reducing Aβ and tau pathology, potentially via nitric oxide signaling and upregulating Aβ transporters such as ABCB1. Lipoic acid [59,60,61,62,63,64,65,66,67,68] shows potent antioxidant and anti-inflammatory properties, mitigating tau hyperphosphorylation, oxidative stress, and behavioral deficits in tauopathy models, while enhancing mitochondrial function. Testosterone Propionate is a synthetic androgen, and androgens are found to regulate tau phosphorylation [69]. Cabazitaxel is a chemotherapy agent. While not directly linked to AD, its ability to stabilize microtubules has prompted interest in its potential to address tau pathology, a hallmark of AD [70]. Probucol is a lipid-lowering drug with antioxidant properties. It may reduce oxidative stress and amyloid deposition, which are implicated in AD [71]. Estradiol valerate and Dienogest are hormonal agents. Estrogen has been studied for its neuroprotective effects, preventing neural tau hyperphosphorylation, particularly in postmenopausal women, who are at higher risk for AD [72]. Perphenazine is an antipsychotic, found, among some others, to lower the levels of insoluble Tau [73]. Degarelix is a GnRH antagonist. Hormonal modulation has been explored in AD, particularly regarding sex hormones and their impact on cognitive function [74]. Isosorbide Dinitrate is a vasodilator. Improving cerebral blood flow may have neuroprotective effects in AD [75]. Ranolazine is an antianginal drug. It modulates cellular metabolism and has been explored for its potential to enhance neuronal energy deficits in AD [76]. Methocarbamol [77], a carbonic anhydrase inhibitor, has been shown to reduce tau toxicity by promoting its clearance. Studies in tauopathy models, including zebrafish and transgenic mice, have demonstrated that methocarbamol can rescue neuronal degeneration, improve cognitive function, and reduce phosphorylated tau levels. Carvedilol is a beta-blocker with antioxidant properties. It may reduce oxidative stress and inflammation, both implicated in AD [78]. Reserpine is an antihypertensive with neuroprotective potential. Reserpine [79,80], in particular, has been studied for its ability to modulate neurotransmitter systems involved in AD. The complete list of the DrugBank candidates is given in the Table S2.

Although not directly involved in interactions with tau protein, the identified drugs affect processes in AD via other targets in the tau signaling pathway, such as phosphorylation. This may be possible due to the PPI interactions in the signaling pathways, occurring at the standard ISM frequency. This suggests the method might capture broader signaling relationships, potentially reflecting in vivo activity, although the mechanism for this requires further investigation and validation.

### 3.2. COCONUT Database Candidates

Compounds from the COCONUT database were also screened on all frequencies, as were the DrugBank compounds. However, in the case of small organic molecules, unlike the proteins, mere calculation of A and *S/N* values cannot be considered the final step. Further insight into the candidate’s structure and properties is required. Therefore, the *dS* values of the candidates were also calculated. Those values were integrated into the final Total Score descriptor as their product, and the candidates were finally sorted accordingly. The top compounds at all frequencies are presented in Table 3.

**Figure 7:**
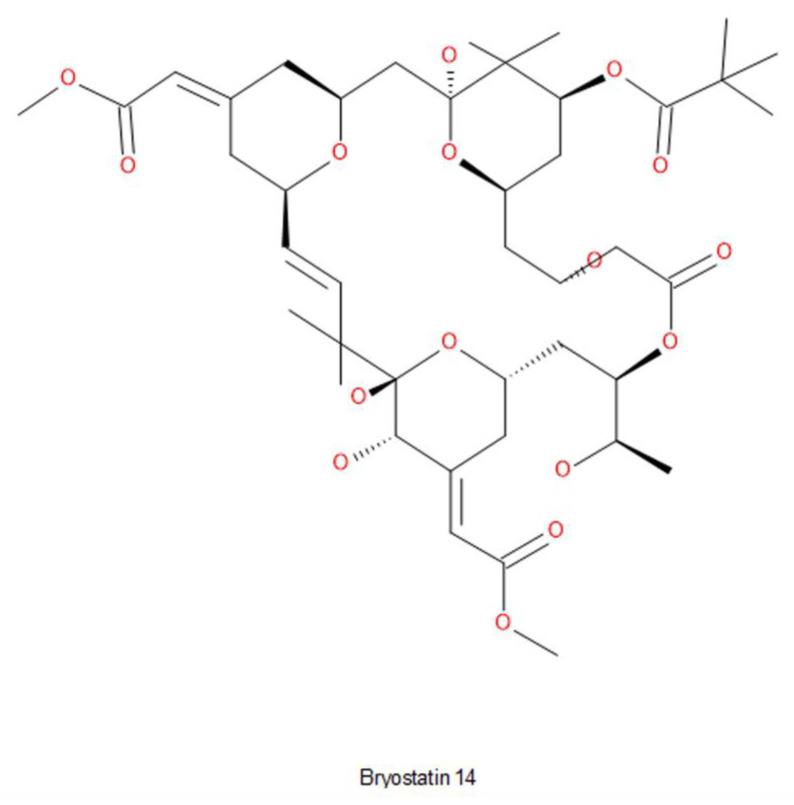
Structure of Bryostatin 14.

We identified a highly ranking compound, bryostatin-14, at F(0.167) (Figure 7). Bryostatins are macrocyclic lactones from marine bryozoans. They are increasingly being considered for therapeutic development in AD because of their ability to modulate protein kinase C (PKC) activity and ultimately mitigate pathological features of AD, such as tau hyperphosphorylation and amyloid-β (Aβ) aggregation. Several preclinical studies have demonstrated that bryostatin-1 enhanced synaptic plasticity and cognitive behavior through the activation of PKCε, which inactivated glycogen synthase kinase-3β (GSK-3β), a critical kinase driving tau hyperphosphorylation [81,82]. The inhibition of GSK-3β reduces the pathological aggregation of tau, which improves neurons’ survival in transgenic tauopathy models [81]. Although their binding to tau protein is not well characterized, their modulation of tau phosphorylation through PKC/GSK-3β signaling makes them an unusual therapeutic approach to targeting tauopathies such as AD. Interestingly, bryostatin-14 is a hit at F(0.167), the most populated F among DrugBank compounds (Figure 6).

### 3.3. Pharmacokinetic Properties of the Selected Compounds

To further assess the druglikeness of the selected compounds, pharmacokinetics calculations were carried out for all three sets of compounds: Tau-interacting drugs (Table 1), DrugBank-identified compounds (Table 2) and COCONUT database candidates (Table 3). ADMET properties were calculated using the pkCSM server [43] (https://biosig.lab.uq.edu.au/pkcsm/prediction, accessed on 25 November 2025), with a total number of variables 36. The resulting data were subjected to k-means clustering.

#### k-Means Clustering

The number of classes was set to 5 and 10 repetitions. The within-class variance was 4.77%, with a between-class variance of 95.23%. Based on the class centroids (Table S2), the five clusters can be interpreted as follows, according to their distinguishing features:

Cluster 1 consists of the smallest molecules (mean molar weight ≈ 410) with the highest lipophilicity (logP ≈ 4.4), BBB permeability (≈−0.28) and CNS permeability (−2.4) among all clusters. These compounds also displayed favorable Caco-2 permeability (0.862), high intestinal absorption (mean value 88%), and high total clearance (logCLtot = 0.571 mL/min/kg). No significant toxicity is predicted, except for hERG II inhibition (around 40% are recognized). The cluster contains the following DrugBank drugs: Astemizole, a direct Tau-interacting compound, Flortaucipir F-18, Lansoprazole, DB01012 (Cinacalcet), DB01393 (Bezafibrate), DB01130 (Prednicarbate), DB01656 (Roflumilast), DB00166 (Lipoic acid), DB01420 (Testosterone Propionate), DB0159 (Probucol), DB08866 (Estradiol valerate), DB00850 (Perphenazine), DB00883 (Isosorbide Dinitrate), DB00243 (Ranolazine), DB00423 (Methocarbamol) and DB01136 (Carvedilol). Fifteen COCONUT candidates belong to this cluster. Regarding best candidates, CNP0327834.1 (#5 rank at F(0.167), Table 3) is the nearest compound to both Astemizol and Lansoprazole. On the other hand, CNP0151916.0 (ranked #8 at F(0.435)) is the nearest one to the DrugBank candidate group in the cluster. The central compound of the cluster is DB01136 (Carvedilol). Members of this group represent the most conventional “drug-like” space.

Cluster 2 contains molecules with intermediate molecular weight (mean MW ≈ 750), moderate lipophilicity (logP ≈ 3.0), and lower BBB permeability characteristics (≈−2.18). These compounds exhibit good intestinal absorption (≈62%) and lower propensity for CNS entry (≈−3.428), although not to the extent of Cluster 1. This cluster includes several clinically approved agents, Docetaxel and Paclitaxel, along with other DrugBank compounds: DB00206 (Reserpine) and DB06772 (Cabazitaxel). Thirteen COCONUT candidates are found in this cluster. CNP0429159.1 (#10 ranked at F(0.435)) is the nearest to Docetaxel, CNP0423521.1 to Pacitaxel, and the central compound of the cluster is CNP0337940.1 (#10 rank at F(0.435)). While still within the expanded drug-like space, diminished BBB and CNS permeability implies primarily peripheral exposure.

Cluster 3 is characterized by higher MW (≈1000) and moderate lipophilicity, similar to cluster 2 (logP ≈ 2.77). Their physicochemical properties indicate lower intestinal absorption, negative Caco2 permeability, and significantly lower BBB (−3.313) and CNS permeability (−4.505) in comparison to Clusters 1 and 2. The compounds also show a higher number of hERG II inhibitors (60%) and higher hepatotoxicity. Total clearance is also significantly lower. This cluster contains two DrugBank compounds, DB06287 (Temsirolimus) and DB01590 (Everolimus). The central compound of the cluster is CNP0180487.0 (#6 ranked at (0.341).

Cluster 4 molecules possess the heaviest mass (MW 1770), extremely low lipophilicity, and extremely low Caco-2 permeability and absorption. Toxicity is on the rise in this cluster. Despite some structural similarity to approved therapeutics, their ADMET profile indicates high development risk. Only one DrugBank compound, DB06699 (Degarelix), belongs to this cluster.

Cluster 5 is the last one, and molecules there show similar properties to those in Cluster 4, with some minor advantages, such as better Caco-2 and BBB permeability, and total clearance, but these values are still far from Clusters 1 and 2. No approved compounds belong to this cluster.

Overall, the k-means analysis separated the dataset into five distinct ADME-driven chemotypes, ranging from CNS-penetrant lipophilic drugs (Cluster 1) to highly complex, permeability-limited compounds (Clusters 4 and 5), with intermediate clusters reflecting balanced or partially drug-like physicochemical profiles. Regarding the COCONUT database candidates, from the table of centroid distances for each cluster, the following best candidates per cluster are presented in Table S2.

**Figure 8:**
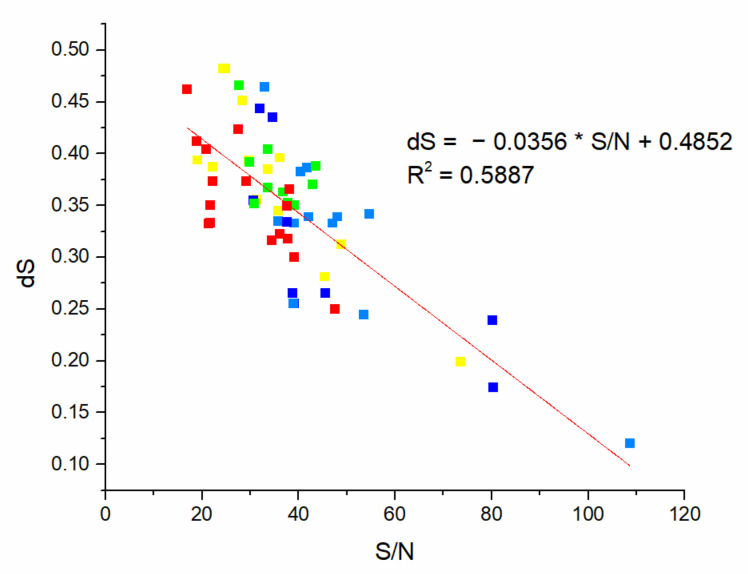
Correlation between *S/N* and Drug Score of COCONUT compounds. The candidates are colored according to k-means clusters. Red: Cluster 1; Yellow: Cluster 2; Green: Cluster 3; Light Blue: Cluster 4; Dark Blue: Cluster 5.

An interesting correlation is found between *S/N* and Drug Score, where R^2^ = 0.589 (Figure 8). This is expected, as *S/N* depends on the amplitude, which, again, depends on the size of the molecule. Larger candidate molecules exhibit a stronger affinity towards proteins. However, the larger the molecule, the less druggable it is, as is also shown in Figure 8. Regardless of F, *S/N* correlates with *dS*. We emphasize that *dS* is the Drug Score, derived from only four properties of a molecule: logP, logS, druggability and molecular weight. Candidates belonging to Cluster 1 (red), the one with best ADMET properties for a small drug molecule, are placed in the area with medium *S/N* values (20–40) and higher *dS* values (0.3–0.45). This suggests that, due to negative correlation between *S/N* and *dS*, a good candidate should compromise both properties in the Total Score.

By combining *S/N* values with distance to centroid in each cluster, we can construct another score—Total Score 2. Since the new score should favor higher *S/N* and lower distance, it should be a ratio of these two values. Therefore, we can rank the compounds per cluster (Table 4). According to the ranking, the candidate with best Total Score 2 is, as already mentioned, CNP0151916.0, a compound with an aporphine structure. It has been reported that aporphines show potential in treating AD, primarily due to their ability to act as cholinesterase inhibitors, similar to some current AD medications. Some aporphines also show promise by reducing oxidative stress and inhibiting β-amyloid formation, which are other key processes in AD [83,84,85,86,87].

### 3.4. Comparison to the Martini-IDP Forcefield

**Figure 9:**
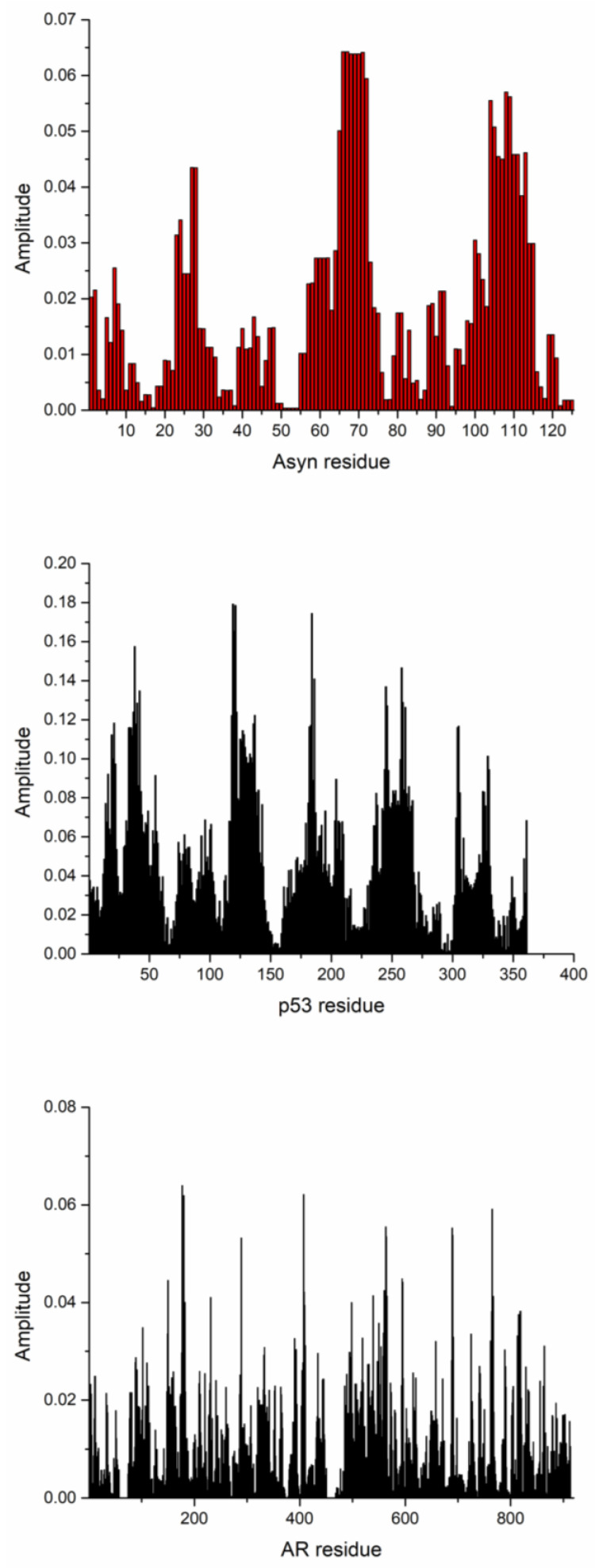
Corresponding amino acid residues in the alpha-synuclein, p53 and adrenergic receptor, at their CS frequencies with the ligands.

In the recent study conducted by Wang et al. [19], the Martini forcefield was updated with the parameters for small molecules. The authors carried out 15 molecular dynamics simulations for the validation. Their study examined the contact frequency between the small molecule–ligand and the IDP. The systems studied were Alpha synuclein, p53, and Androgen receptor, as well as their corresponding ligands (see [19]). The corresponding domains with the highest contribution to protein–ligand ISM-SM CS frequencies (Figure 9), and their comparison to the Martini-IDP forcefield, are presented in Table 5.

The results demonstrate that ISM-SM complements traditional forcefield-based approaches, offering alternative insights into IDP-ligand binding regions. The observed overlaps support ISM-SM’s validity, while the method’s broader residue selection suggests it could be particularly valuable for capturing dynamic, transient interactions—hallmarks of intrinsically disordered proteins.

### 3.5. Comparison to Ensemble Docking Results

**Figure 10:**
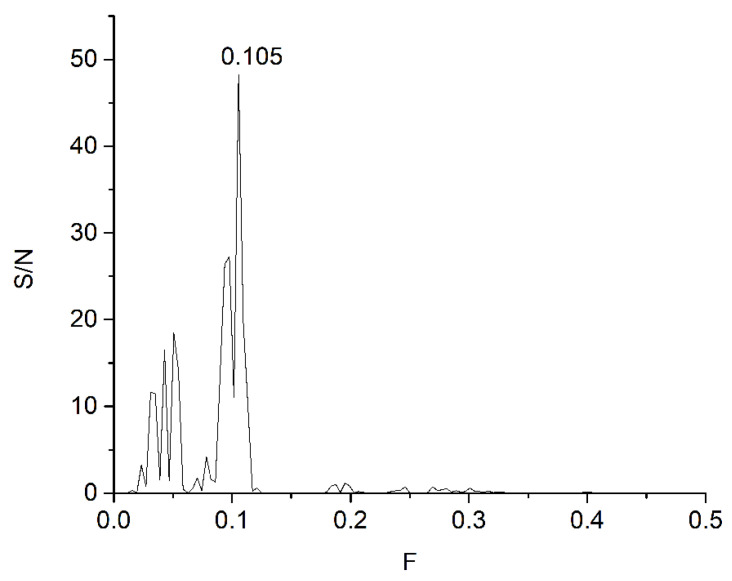
Cross-spectrum of alpha-synuclein and all three targeting ligands.

A similar study on the ensemble docking of three compounds to alpha-synuclein has been reported recently [7]. Fasudil, Ligand 47 and Ligand 23 were docked to a-synuclein conformations obtained from the MD simulations. According to the results, the amino acid residues with the highest probability for interaction with the ligands are in the domain 121–139, with the top values at Y133 and E137. The ISM-SM analysis confirms this. In Table 6 are presented the top three peaks for each protein–ligand interaction, and corresponding protein domains from the slide window analysis. Finally, Figure 10 presents the common frequency from the CS of the protein and all three ligands, as well as their CS.

**Figure 11:**
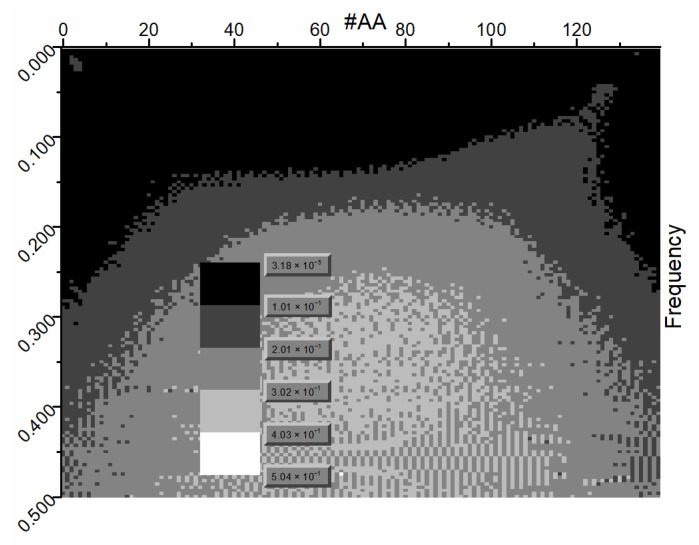
CWT plot of alpha-synuclein at all ISM frequencies. The color scale of CWT coefficients is incorporated in the figure.

**Figure 12:**
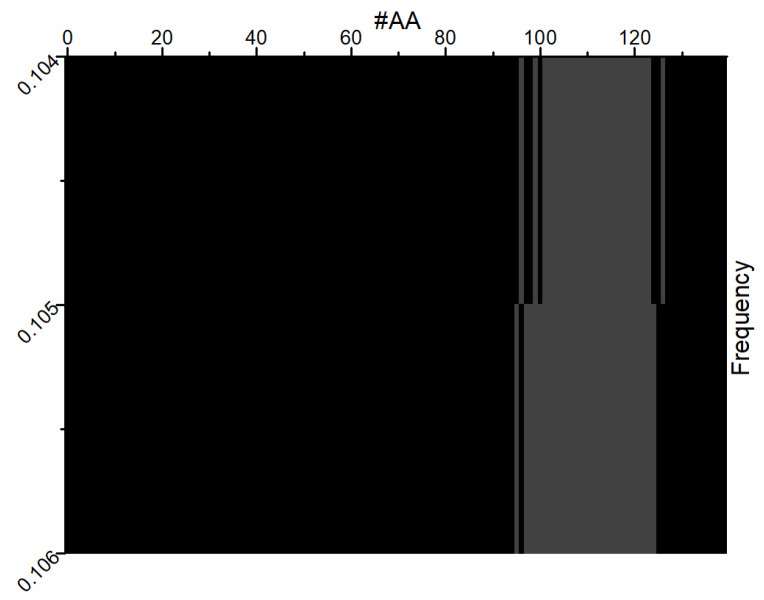
CWT heatmap of alpha-synuclein at ISM frequency F(0.105).

To narrow the interaction domain and identify interaction hotspots, a continuous wavelet transform of the alpha-synuclein sequence was performed, similar to our previous study [44]. The heatmaps of direct wavelet coefficient values are presented in Figure 11 and Figure 12, in five shades. Notably, the “hotspot” amino acid residues are in the range of 100–122 (Figure 12). This is in good accordance with the ensemble docking results. Therefore, the ISM-SM method can successfully predict and interpret interaction domains of protein–ligand pairs, based on their sequences, as effectively as structure-based methods.

## 4. Conclusions

In this study, we examined the applicability and reliability of the ISM-SM method in identifying small-molecule candidates relevant to IDPs, taking human tau protein as a test case. Unlike many other methods, ISMSM does not assume predefined 3D structures but relies on EIIP-based spectral compatibility in order to detect long-range interaction features. Although conceptually different from structure-based approaches, several lines of evidence are presented showing that the method yields biologically meaningful results.

First, ISM-SM recovered a set of approved drugs already reported to modulate tau pathology or Alzheimer’s-related signaling pathways. Although many of these compounds act indirectly, their consistent enrichment at characteristic ISM frequencies supports the method’s ability to capture functionally relevant interaction signatures. Second, the identified natural-product candidates, such as bryostatin-14 and aporphines, show coherence with known tau-related pathways, further supporting the biological relevance of ISM frequencies. Third, comparison with Martini-IDP simulations and ensemble docking revealed overlaps in residues contributing to interaction hotspots. These findings suggest that ISM-SM captures elements of the dynamic recognition patterns typical for IDPs, complementing force-field- and structure-based methods.

At the same time, it is important to recognize the limitations of the method. First, ISM-SM predicts compatibility in the informational spectrum rather than physical binding, and thus cannot directly confirm binding affinity, binding pose, or conformational stabilization. Its use is therefore most reliable as an early-stage screening and hypothesis-generation tool, with experimental validation or detailed structural simulations remaining essential for mechanism-level interpretation. Furthermore, while the ability of ISM-SM to identify compounds acting indirectly through signaling pathways may be an advantage, careful interpretation and further theoretical clarification are required.

Overall, the current results position ISM-SM as a robust and computationally efficient complementary method to explore chemical space around IDP targets. Its strengths include screening large databases at high speeds and the identification of conserved interaction frequencies. Prioritization of candidates based on their favorable pharmacokinetic properties can be easily implemented. Appropriately, together with structural, biophysical, and machine learning methods, ISM-SM can expand the toolbox currently available for rational ligand discovery against dynamic and structurally heterogeneous protein targets.

## Figures and Tables

**Table 1:** Tau-interacting compounds from the DrugBank, with corresponding frequencies from CS with the tau protein.

DrugBank Compound	Name	CS with tau Frequencies	Amplitude	S/N	Corresponding Domain in the tau Protein	Literature Binding Region
DB00637	Astemizole	0.342	0.9958	17.353	269–525	386–391
DB01248	Docetaxel	0.167	1.7847	20.023	62–318	β-tubulin
DB14914	Flortaucipir F-18	0.080	0.10816	9.0225	427–683	R3–R4 386–391
DB00448	Lansoprazole	0.435	0.65514	12.299	305–561	R3–R4 386–391
DB01229	Paclitaxel	0.194	1.1917	14.089	23–279	β-tubulin

**Table 2:** The list of identified DrugBank compounds from the tau protein ISM spectrum.

No	ID	Name	Amplitude	S/N	Frequency	Effect on tau Protein/AD
1	DB01012	Cinacalcet	0.40481	13.45278	0.080	Indirect on tau phosphorylation
2	DB01393	Bezafibrate	0.32723	9.6733	0.080	Reduces Aβ and tau pathology
3	DB06287	Temsirolimus	1.14833	28.35191	0.167	Reducing tau hyperphosphorylation
4	DB01590	Everolimus	3.23776	20.74836	0.167	Reducing tau hyperphosphorylation
5	DB00035	Desmopressin	2.30313	20.55908	0.167	Could influence Aβ/tau cross-interactions
6	DB01130	Prednicarbate	1.34609	18.58936	0.167	Potential tau aggregation modulator
7	DB01656	Roflumilast	17.85085	0.05962	0.167	Ameliorates cognitive deficits in tauopathy models
8	DB00166	Lipoic acid	17.1443	0.04087	0.167	Reduces tauopathy
9	DB01420	Testosterone Propionate	1.00595	22.21979	0.194	Hyperphosphorylation of tau
10	DB06772	Cabazitaxel	2.47515	22.12693	0.194	Microtubule stabilization
11	DB01599	Probucol	0.50271	19.55832	0.194	Reduce amyloid deposition
12	DB08866	Estradiol valerate/Dienogest	0.91072	19.02854	0.194	Prevents tau hyperphosphorylation
13	DB00850	Perphenazine	0.71948	14.65717	0.333	Lower the levels of insoluble tau.
14	DB06699	Degarelix	2.05541	13.78509	0.333	Hormone modulation may influence neurodegeneration
15	DB00883	Isosorbide Dinitrate	0.87453	22.00889	0.342	Nitric oxide modulation (could influence neurodegeneration)
16	DB00243	Ranolazine	1.48002	23.2534	0.435	Reduces oxidative stress, lacks tau-specific evidence
17	DB00423	Methocarbamol	0.80441	21.58701	0.435	Promoting tau clearance
18	DB01136	Carvedilol	1.07707	21.14359	0.435	May reduce Aβ and tau toxicity
19	DB00206	Reserpine	2.15401	19.96937	0.435	Reduces Aβ toxicity

**Table 3:** The top ten compounds from the COCONUT database, at all frequencies, ranked by Total Score. Bryostatin-14 is printed in bold.

Compound ID	Amplitude	S/N	Drug Score	Total Score	F
CNP0504067.0	2.6931	27.5146	0.4236	11.6548	0.080
CNP0126636.1	2.0462	22.2079	0.3871	8.5975	
CNP0560502.0	0.9447	20.8465	0.4040	8.4210	
CNP0195295.1	1.7374	22.2690	0.3734	8.3160	
CNP0532732.0	0.5916	16.8757	0.4619	7.7952	
CNP0581434.0	0.9140	18.8845	0.4116	7.7720	
CNP0111317.1	1.0835	21.7010	0.3503	7.6024	
CNP0154283.1	1.7748	19.1053	0.3937	7.5220	
CNP0285595.1	0.9112	21.7744	0.3330	7.2507	
CNP0511033.0	0.7804	21.4297	0.3323	7.1204	
CNP0266316.1	4.2790	33.7220	0.3849	12.9806	0.167
**CNP0168057.1**	**4.9895**	**35.8560**	**0.3450**	**12.3714**	
CNP0427543.1	3.5080	37.8452	0.3181	12.0369	
CNP0135438.1	3.0234	39.1900	0.3002	11.7653	
CNP0327834.1	2.5014	36.2093	0.3224	11.6753	
CNP0297394.1	3.1593	31.3039	0.3558	11.1366	
CNP0359990.1	3.0488	34.4367	0.3164	10.8969	
CNP0449680.1	6.3795	30.7200	0.3544	10.8859	
CNP0072358.1	1.5625	29.1654	0.3732	10.8849	
CNP0280000.1	4.7052	30.8741	0.3516	10.8550	
CNP0267855.1	7.1527	54.7695	0.3416	18.7103	0.194
CNP0115161.1	5.9639	48.0360	0.3389	16.2787	
CNP0271940.1	6.0078	47.1667	0.3328	15.6975	
CNP0144759.1	3.8288	32.9508	0.4640	15.2877	
CNP0399889.1	5.2859	42.0429	0.3389	14.2478	
CNP0271195.1	5.0978	39.0668	0.3328	13.0018	
CNP0206347.1	5.2161	27.6665	0.4660	12.8917	
CNP0075233.1	4.1613	35.8159	0.3348	11.9926	
CNP0199404.1	2.0896	47.5628	0.2497	11.8770	
CNP0337940.1	3.9174	29.6884	0.3930	11.6670	
CNP0425508.1	8.0539	41.7191	0.3861	16.1071	0.333
CNP0426456.1	7.3556	40.4095	0.3824	15.4512	
CNP0580557.0	79.8827	108.7010	0.1205	13.0938	
CNP0492610.1	6.6601	45.5939	0.2655	12.1062	
CNP0574550.1	4.4607	24.8369	0.4820	11.9716	
CNP0493035.1	4.3748	24.4605	0.4820	11.7901	
CNP0598400.0	5.2834	29.8102	0.3916	11.6749	
CNP0571478.1	5.5340	38.7741	0.2655	10.2954	
CNP0491847.1	5.6254	39.1878	0.2556	10.0162	
CNP0357360.0	7.9691	39.0120	0.2556	9.9713	
CNP0285895.1	8.6899	43.5842	0.3877	16.8988	0.341
CNP0313376.1	8.3450	42.9529	0.3704	15.9113	
CNP0578185.1	6.3962	32.0514	0.4437	14.2199	
CNP0291861.1	6.2579	39.1064	0.3501	13.6928	
CNP0538593.1	3.9683	33.6090	0.4039	13.5734	
CNP0180487.0	5.7451	36.7896	0.3628	13.3468	
CNP0525297.1	6.1290	37.8186	0.3521	13.3144	
CNP0423521.1	5.3032	45.4780	0.2811	12.7837	
CNP0549106.1	7.1238	37.6706	0.3341	12.5872	
CNP0319138.1	4.8550	33.6416	0.3671	12.3481	
CNP0551487.1	16.0579	80.2087	0.2389	19.1640	0.435
CNP0199424.0	6.8985	48.9195	0.3121	15.2669	
CNP0509389.2	3.9253	34.6086	0.4348	15.0463	
CNP0105199.1	15.3904	73.5380	0.1991	14.6407	
CNP0417346.0	4.4444	36.2080	0.3960	14.3374	
CNP0048849.1	26.4727	80.3243	0.1742	13.9922	
CNP0061932.1	2.4242	38.1590	0.3657	13.9535	
CNP0151916.0	2.4107	37.6099	0.3492	13.1339	
CNP0078724.1	20.8988	53.4832	0.2448	13.0907	
CNP0429159.1	1.9277	28.3867	0.4510	12.8016	

**Table 4:** The top compounds from the COCONUT database per cluster, ranked by Total Score 2. Bryostatin-14 and CNP0151916.0 are printed in bold.

Compound ID	S/N	Drug Score	Distance to Cluster	F	Total Score 2 (S/N/Distance to Centroid)	Cluster
**CNP0151916.0**	**37.6099**	**0.3492**	**23.1768**	**0.435**	**1.6227**	1
CNP0327834.1	36.2093	0.3224	53.6144	0.167	0.6754	
CNP0072358.1	29.1654	0.3732	44.1454	0.167	0.6607	
CNP0504067.0	27.5146	0.4236	54.1343	0.08	0.5083	
CNP0199404.1	47.5628	0.2497	110.9528	0.194	0.4287	
CNP0061932.1	38.1590	0.3657	101.6911	0.435	0.3752	
CNP0427543.1	37.8452	0.3181	103.7778	0.167	0.3647	
CNP0135438.1	39.1900	0.3002	132.4314	0.167	0.2959	
CNP0511033.0	21.4297	0.3323	75.7621	0.08	0.2829	
CNP0560502.0	20.8465	0.4040	79.2396	0.08	0.2631	
CNP0111317.1	21.7010	0.3503	97.8961	0.08	0.2217	
CNP0359990.1	34.4367	0.3164	164.0605	0.167	0.2099	
CNP0581434.0	18.8845	0.4116	94.3867	0.08	0.2001	
CNP0285595.1	21.7744	0.3330	110.4145	0.08	0.1972	
CNP0532732.0	16.8757	0.4619	105.8951	0.08	0.1594	
CNP0195295.1	22.2690	0.3734	173.7049	0.08	0.1282	
CNP0337940.1	29.6884	0.3930	15.2877	0.194	1.9420	2
CNP0199424.0	48.9195	0.3121	44.9265	0.435	1.0889	
CNP0574550.1	24.8369	0.4820	26.6104	0.333	0.9334	
CNP0493035.1	24.4605	0.4820	26.3254	0.333	0.9292	
CNP0266316.1	33.7220	0.3849	44.7305	0.167	0.7539	
CNP0126636.1	22.2079	0.3871	35.9000	0.08	0.6186	
CNP0105199.1	73.5380	0.1991	140.4245	0.435	0.5237	
**CNP0168057.1**	**35.8560**	**0.3450**	**78.0769**	**0.167**	**0.4592**	
CNP0429159.1	28.3867	0.4510	64.1686	0.435	0.4424	
CNP0154283.1	19.1053	0.3937	46.2699	0.08	0.4129	
CNP0423521.1	45.4780	0.2811	115.1818	0.341	0.3948	
CNP0417346.0	36.2080	0.3960	107.3436	0.435	0.3373	
CNP0297394.1	31.3039	0.3558	95.0548	0.167	0.3293	
CNP0180487.0	36.7896	0.3628	13.9520	0.341	2.6369	3
CNP0313376.1	42.9529	0.3704	20.4250	0.341	2.1030	
CNP0525297.1	37.8186	0.3521	34.1682	0.341	1.1068	
CNP0319138.1	33.6416	0.3671	44.9490	0.341	0.7484	
CNP0291861.1	39.1064	0.3501	66.2943	0.341	0.5899	
CNP0285895.1	43.5842	0.3877	79.0845	0.341	0.5511	
CNP0598400.0	29.8102	0.3916	57.8419	0.333	0.5154	
CNP0280000.1	30.8741	0.3516	77.4212	0.167	0.3988	
CNP0538593.1	33.6090	0.4039	99.8643	0.341	0.3365	
CNP0206347.1	27.6665	0.4660	87.9175	0.194	0.3147	
CNP0115161.1	48.0360	0.3389	64.0638	0.194	0.7498	4
CNP0271940.1	47.1667	0.3328	63.0634	0.194	0.7479	
CNP0399889.1	42.0429	0.3389	64.2465	0.194	0.6544	
CNP0271195.1	39.0668	0.3328	62.7261	0.194	0.6228	
CNP0267855.1	54.7695	0.3416	112.7427	0.194	0.4858	
CNP0075233.1	35.8159	0.3348	78.4168	0.194	0.4567	
CNP0580557.0	108.7010	0.1205	266.9120	0.333	0.4073	
CNP0426456.1	40.4095	0.3824	151.0078	0.333	0.2676	
CNP0425508.1	41.7191	0.3861	166.1188	0.333	0.2511	
CNP0357360.0	39.0120	0.2556	163.7966	0.333	0.2382	
CNP0078724.1	53.4832	0.2448	356.3395	0.435	0.1501	
CNP0144759.1	32.9508	0.4640	224.4241	0.194	0.1468	
CNP0551487.1	80.2087	0.2389	134.2948	0.435	0.5973	5
CNP0048849.1	80.3243	0.1742	137.0805	0.435	0.5860	
CNP0509389.2	34.6086	0.4348	79.4140	0.435	0.4358	
CNP0492610.1	45.5939	0.2655	121.3631	0.333	0.3757	
CNP0491847.1	39.1878	0.2556	106.6358	0.333	0.3675	
CNP0549106.1	37.6706	0.3341	108.1871	0.341	0.3482	
CNP0578185.1	32.0514	0.4437	95.4639	0.341	0.3357	
CNP0571478.1	38.7741	0.2655	121.3645	0.333	0.3195	
CNP0449680.1	30.7200	0.3544	102.7158	0.167	0.2991	

**Table 5:** The key residues for IDP–ligand interactions were compared from MD simulations with the Martini-IDP forcefield and the ISM-SM method. The residues most approximately identified in both methods are in bold.

System	Martini-IDP	ISM-SM Region
alpha-synuclein- fasudil	**3**, 38, **93**, **115**, 124, 127, 135	**1**, **2**, 7, 23, 24, 27, 28, 66, 67, 68, 69,70, 71, **104**, 108, **109** (Slide window width 17)
p53—Ligand 1050	**23**	**21**, 38, 119, 184, 258, 305, 329, 361 (Slide window width 33)
AR—EPI-002	396, **405**, **406**, 432, 433, 437, 438	177, 289, **407**, 563, 689, 765 (Slide window width 8)

**Table 6:** Top peaks for each alpha-synuclein–ligand interaction, for all three ligands’ common interaction and corresponding protein domains from slide window analysis.

Protein–Ligand Complex	Peak	F	A	S/N	ISM-SM Region
Ligand47	1	0.054	0.0772	11.18	42–106
	2	0.105	0.0615	8.9147	4–132
	3	0.226	0.0438	6.3474	70–134
Fasudil	1	0.105	0.0972	12.359	4–132
	2	0.031	0.0720	9.1587	57–121
	3	0.093	0.0667	8.4796	7–71
Ligand23	1	0.300	0.0342	7.4993	57–121
	2	0.351	0.0298	6.5445	71–135
	3	0.105	0.0287	6.2984	4–132
All three ligands	1	0.105	0.0003	48.2984	4–132

## Data Availability

This article and the Supplementary Material include the original contributions presented in this study. Further inquiries can be directed to the corresponding author.

## Supplementary Materials

The following supporting information can be downloaded at: https://www.mdpi.com/article/10.3390/mps8060150/s1. Table S1: contains the lists of DrugBank and COCONUT database candidates at all ISM frequencies, along with the amino acid and atomic group EIIP parameters used in the calculations. Table S2: contains the ADMET properties of all compounds, k-means clustering and their ranking by Total Score 2.

## Abbreviations

The following abbreviations are used in this manuscript:

IDP	Intrinsically disordered protein
EGCG	Epigallocatechin gallate
MD	Molecular Dynamics
NMR	Nuclear Magnetic Resonance
SPR	Surface Plasmon Resonance
ISM-SM	Informational Spectrum Method for Small Molecules
HTPS	High-Throughput Screening
AD	Alzheimer’s disease
EIIP	Electron Ion Interaction Potential
DFT	Discrete Fourier Transformation
IS	Informational Spectra
CS	Cross Spectrum
MW	Molecular Weight
dS	Drug Score
S/N	Signal-to-Noise ratio
MTBD	Microtubule-Binding
Aβ	Amyloid-Beta
PKC	Protein Kinase C
GSK	Glycogen Synthase Kinase
CWT	Continuous Wavelet Transform
